# Atelectasis causes alveolar hypoxia-induced inflammation during uneven mechanical ventilation in rats

**DOI:** 10.1186/s40635-015-0056-z

**Published:** 2015-06-19

**Authors:** Kentaro Tojo, Yusuke Nagamine, Takuya Yazawa, Takahiro Mihara, Yasuko Baba, Shuhei Ota, Takahisa Goto, Kiyoyasu Kurahashi

**Affiliations:** Department of Anesthesiology and Critical Care Medicine, Yokohama City University Graduate School of Medicine, 3-9, Fukuura, Kanazawa-ku, Yokohama-city, Kanagawa 236-0004 Japan; Department of Diagnostic Pathology, Chiba University Graduate School of Medicine, 1-8-1, Inohana, Chuo-ku, Chiba-city, Chiba 260-8670 Japan; Department of Anesthesiology, Kanagawa Children’s Medical Center, 2-138-4, Mutsukawa, Minami-ku, Yokohama-city, Kanagawa 232-8555 Japan; Operation Department, Yokohama City University Medical Center, 4-57, Urafune, Minami-ku, Yokohama-city, Kanagawa 232-0024 Japan; Department of Palliative Care Medicine, Kanagawa Cancer Center, 2-3-2, Nakao, Asahi-ku, Yokohama-city, Kanagawa 241-8515 Japan

**Keywords:** Atelectasis, Alveolar hypoxia, Ventilator-associated lung injury, Proinflammatory cytokines, Hypoxia inducible factor-1, Nuclear factor-κB

## Abstract

**Background:**

Patients with acute respiratory distress syndrome receiving mechanical ventilation show inhomogeneous lung aeration. Atelectasis during uneven mechanical ventilation leads to alveolar hypoxia and could therefore result in lung inflammation and injury. We aimed to elucidate whether and how atelectasis causes alveolar hypoxia-induced inflammation during uneven mechanical ventilation in an open-chest differential-ventilation rat model.

**Methods:**

We first investigated inflammatory and histological changes in the bilateral lungs of unilaterally ventilated rats, in which the right lung was atelectatic and the left lung was ventilated with high tidal volume (HTV). In the next series, we investigated the effects of normal tidal volume (NTV) ventilation of the right lungs with 60 % O_2_ or 100 % N_2_ during HTV ventilation of the left lungs. Then, proinflammatory cytokine secretions were quantified from murine lung epithelial (MLE15) and murine alveolar macrophage (MH-S) cells cultured under a hypoxic condition (5 % O_2_) mimicking atelectasis. Further, activities of nuclear factor (NF)-κB and hypoxia-inducible factor (HIF)-1 were assessed in the nonventilated atelectatic lung and MLE15 cells cultured under the hypoxic condition. Finally, effects of NF-κB inhibition and HIF-1α knockdown on the cytokine secretions from MLE15 cells cultured under the hypoxic condition were assessed.

**Results:**

The nonventilated atelectatic lungs showed inflammatory responses and minimal histological changes comparable to those of the HTV-ventilated lungs. NTV ventilation with 60 % O_2_ attenuated the increase in chemokine (C-X-C motif) ligand (CXCL)-1 secretion and neutrophil accumulation observed in the atelectatic lungs, but that with 100 % N_2_ did not. MLE15 cells cultured with tumor necrosis factor (TNF)-α under the hypoxic condition showed increased CXCL-1 secretion. NF-κB and HIF-1α were activated in the nonventilated atelectatic lungs and MLE15 cells cultured under the hypoxic condition. NF-κB inhibition abolished the hypoxia-induced increase in CXCL-1 secretion from MLE15 cells, while HIF-1α knockdown augmented it.

**Conclusions:**

Atelectasis causes alveolar hypoxia-induced inflammatory responses including NF-κB-dependent CXCL-1 secretion from lung epithelial cells. HIF-1 activation in lung epithelial cells is an anti-inflammatory response to alveolar hypoxia in atelectatic lungs.

## Background

Patients with acute respiratory distress syndrome receiving mechanical ventilation show inhomogeneous lung aeration. Dependent atelectatic regions are poorly aerated while nondependent aerated regions are ventilated with relatively high tidal volume (HTV). HTV ventilation of nondependent regions causes overinflation and barotrauma [1]. Moreover, peri-atelectatic regions augment mechanical stress [2], and repetitive shear stress caused by alveolar collapse and re-opening deteriorates lung injury [3–5]. Notably, alveolar O_2_ tension rapidly decreases to a mixed venous level in atelectatic lungs [6], leading to alveolar hypoxia, a potent inducer of lung inflammation [7]. Therefore, atelectasis could deteriorate ventilator-associated lung injury (VALI) by alveolar hypoxia-induced inflammation during uneven mechanical ventilation. However, the role of atelectasis in VALI is not fully elucidated.

Nuclear factor (NF)-κB and hypoxia-inducible factor (HIF)-1 are transcription factors that regulate hypoxic inflammation [8, 9]. NF-κB has a central role in the transcription of proinflammatory mediators, responsible for inflammatory diseases including lung injury [10]. It is inactive when bound to inhibitors of κB (IκB) in the cytoplasm, but IκB phosphorylation by IκB kinase leads to liberation and nuclear translocation of NF-κB. Hypoxia activates IκB kinase through inhibition of prolyl hydroxylase (PHD)-1 and enhances nuclear translocation of NF-κB [11]. HIF-1 is a heterodimer consisting of HIF-1α and HIF-1β. In normoxic conditions, specific proline residues of HIF-1α are hydroxylated by PHD and HIF-1α is ubiquitinated and degraded, while HIF-1β is constitutively present in excess. Hypoxia inhibits HIF-1α hydroxylation by PHD and activates HIF-1. HIF-1 is a key regulator of hypoxic responses and modulates transcription of numerous genes associated with adaptation to hypoxia [12]. It also plays essential roles in regulating inflammation [13–17].

To elucidate whether and how atelectasis causes alveolar hypoxia-induced inflammation, we used an open-chest differential-ventilation rat model. First, we investigated inflammation and histological changes in the bilateral lungs of unilaterally ventilated rats, in which the right lung was atelectatic and the left lung was ventilated with HTV. Next, we investigated the effects of normal tidal volume (NTV) ventilation of the right lungs with 60 % O_2_ (nonhypoxic condition) or 0 % O_2_ (hypoxic condition) during HTV ventilation of the left lungs. Then, we studied the responses of two potent sources of inflammatory mediators in the lungs: lung epithelial and alveolar macrophage cells, under a hypoxic condition mimicking atelectasis. To clarify the mechanisms of lung injury, we investigated the roles of NF-κB and HIF-1 in hypoxia-induced inflammatory responses in lung epithelial cells.

## Methods

### Animals

The Animal Research Committee of Yokohama City University approved the animal experiments. Specific pathogen-free male Sprague–Dawley rats aged 9–10 weeks and weighing 300–400 g were used. The animals were housed under a 12-h light/dark cycle with unrestricted access to food and water.

### Ventilation protocols

The animals were anesthetized with an intraperitoneal injection of sodium pentobarbital (16 mg), and 0.5 % bupivacaine was injected into the surgical site for analgesia. The right carotid artery and femoral vein were catheterized. Lactated Ringer’s solution containing 0.5 % sodium pentobarbital was continuously injected (4 mL/kg/h) via the right femoral vein. Body temperature was maintained between 36.5 and 38.0 °C by placing the rats on a warming device. After tracheotomy, a 16-gauge plastic cannula processed with a broadened tip was inserted into the trachea. After administration of 1-mg pancuronium bromide and initiation of mechanical ventilation, bilateral thoracotomy was performed to visualize the lungs.

Rats were assigned to one of two ventilation protocols (eight animals per protocol): bilateral lung ventilation (BLV) and unilateral lung ventilation (ULV). BLV was initiated and maintained by using a constant-volume pump (SN-480-7, Shinano Seisakusho, Tokyo, Japan) with the following parameters: tidal volume, 8 mL/kg; frequency, 80/min; and positive end-expiratory pressure (PEEP), 4 cmH_2_O. For ULV, the 16-gauge tracheal cannula was advanced into the left main bronchus and the left lung was ventilated with the same parameter setting for BLV: tidal volume, 8 mL/kg; frequency, 80/min; and PEEP, 4 cmH_2_O. Immediately thereafter, a 20-gauge plastic cannula was inserted into the trachea through the tracheotomy site in the ULV group. The gap between the cannula and the trachea was sealed with instant adhesive (Aron Alpha, Toagosei, Tokyo, Japan) to prevent air leakage. The 20-gauge tracheal cannula was open to atmospheric pressure, and the right lung was collapsed. The inspired O_2_ fraction of the ventilated lungs was 1.0 during the first hour to prevent severe hypoxemia before establishment of hypoxic pulmonary vasoconstriction and then reduced to 0.6. Before and every 1 h after the ventilation protocols were applied, the ventilated lungs were inflated at 30 cmH_2_O for 5 s to prevent atelectasis.

In the next series of experiments, we evaluated the effects of NTV ventilation of right lungs with different gaseous mixtures during HTV ventilation of the left lungs. Rats were assigned to one of three ventilation protocols: ULV, NTV60 %, and NTV0 %. In all the groups, the left lung was ventilated through the 16-gauge cannula in the left main bronchus with the following parameters: tidal volume, 8 mL/kg; frequency, 80/min; and PEEP, 4 cmH_2_O. The ventilation protocol of the ULV group was the same to that of the first series experiment. For NTV ventilation of the right lung, the 20-gauge tracheal cannula was connected to another constant-volume pump and the right lung was ventilated with the following parameters: tidal volume, 4 mL/kg; frequency, 80/min; and PEEP, 4 cmH_2_O. The inspired O_2_ fraction of the right NTV lung was either 60 or 0 % O_2_ (100 % N_2_).

Mean arterial pressure (MAP) and peak airway pressure (*P*_aw_) were recorded, and arterial blood was collected for blood gas analysis before and every 2 h after the ventilation protocols, and the volume of blood obtained was replaced with the same volume of 6 % hydroxyethyl starch solution (Hespander, Fresenius Kabi Japan, Tokyo, Japan). After 8 h of ventilation, the rats were deeply anesthetized and euthanized by exsanguination from the right carotid artery. Their pulmonary vessels were flushed with 50 mL of ice-cold normal saline containing 0.3-mM ethylenediaminetetraacetic acid by gravity infusion at 30 cmH_2_O via the right ventricle, and the lungs were excised.

### Cell culture

Murine lung epithelial (MLE15) and murine alveolar macrophage (MH-S) cells were plated on 12-well plates (2.5 × 10^5^ cells per well ) and cultured in RPMI-1640 (Nacalai Tesque, Tokyo, Japan) supplemented with 4 and 10 % fetal bovine serum (FBS), respectively, for 24 h. The cells were then FBS-starved for 12 h. Subsequently, the culture medium was exchanged to FBS-free medium with or without 500 pg/mL tumor necrosis factor (TNF)-α (PeproTech, Rocky Hill, NJ) to simulate a sterile inflammatory environment, and the cells were cultured in either 21 % (nonhypoxic condition) or 5 % (hypoxic condition) O_2_ by using CulturePal5 (Mitsubishi Gas Chemical Company, Tokyo, Japan) for 24 h. Conditioned medium was collected and centrifuged at 3000*g* for 10 min at 4 °C. The supernatants were aliquoted and stored at −80 °C until use. Finally, the cells were lysed for either protein or RNA extraction.

### Histopathology

Lung tissues were fixed in paraformaldehyde and embedded in paraffin as described previously [18], and their sections were stained with hematoxylin and eosin. A pathologist blinded to the allocation of the ventilation protocols assessed and scored the degree of lung histological changes. Perivascular and peribronchial edema, infiltration of leukocytes into the alveolar spaces, and leukocyte stasis and attachment to the intima of the vascular walls were independently scored as 0, none; 1, mild-to-moderate; or 2, severe. The sum of each score (range, 0 to 6) was defined as the histological score.

### ELISA

Frozen lung blocks were homogenized in 10 volumes of phosphate-buffered saline containing 0.5 % Triton-X and 1.0 % proteinase inhibitor cocktail (25954–21, Nacalai Tesque) on ice and centrifuged at 10,000*g* for 10 min at 4 °C. The supernatants were aliquoted and stored at −80 °C until use. The concentrations of TNF-α (DY510, R&D Systems, Minneapolis, MN), chemokine (C-X-C motif) ligand (CXCL)-1 (DY515, R&D Systems), chemokine (C-C motif) ligand (CCL)-2 (900-M59, PeproTech), and myeloperoxidase (MPO) (DY3667, R&D Systems) were determined by enzyme-linked immunosorbent assay (ELISA). The values were normalized to the total protein concentration measured by a BCA protein assay kit (Thermo Fisher Scientific, Yokohama, Japan).

CXCL-1 (DY453, R&D Systems), CCL-2 (900-K126, PeproTech), and TNF-α (DY410, R&D Systems) in cell-culture supernatants were quantified by ELISA according to the manufacturers’ instructions. Each cytokine concentration was normalized to the relative cell density determined by naphthol blue-black staining.

For HIF-1, lung tissues were homogenized in RIPA buffer (ab156034, Abcam, Cambridge, UK) and sonicated on ice. Cultured cells were washed with ice-cold PBS twice and lysed with ice-cold RIPA buffer for 1 h on ice. Lung tissue homogenates and cell lysates were centrifuged at 10,000*g* for 10 min at 4 °C, and HIF-1α in the supernatants was quantified by ELISA (DY-1935, R&D Systems).

### NF-κB p65 binding activity assay

The NF-κB p65 binding activities of whole cell lysates of lung tissues were quantified by ELISA (TransAM NFκB p65, Active Motif, Carlsbad, CA). Those of nuclear proteins extracted from cultured cells by using the NE-PER Nuclear and Cytoplasmic Extraction Reagent Kit (Thermo Fisher Scientific) were also quantified by ELISA (10007889, Cayman Chemical, Ann Arbor, MI). The activities were normalized to the total protein concentration.

### Reverse transcription-qPCR

RNA was extracted from frozen lung blocks by using Sepasol-RNA Super G (Nacalai Tesque) and from cultured cells by using RNA extraction columns (NucleoSpin RNA II, Takara Bio, Shiga, Japan). Reverse transcription-PCR was performed to obtain cDNA. Then, reverse transcription-quantitative polymerase chain reaction (qPCR) was performed by using SYBR Premix ExTaq (Takara Bio) with specific primers (Life Technologies Japan, Tokyo, Japan) (Table 1) under the following conditions: 30 s at 95 °C and 40 cycles for 5 s at 95 °C and 30 s at 60 °C (iCycler, Bio-Rad Laboratories, Hercules, CA). Changes in *HIF1A*, vascular endothelial growth factor A (*VEGFA*), and glucose transporter-1 (*GLUT1*) mRNA expressions relative to β-actin (*ACTB*) mRNA expression were calculated.

**Table 1:** Primers for qPCR

Gene	Primer	Sequence
Mouse *HIF1A*	Forward	5′-ATCAAGTCAGCAACGTGGAA-3′
	Reverse	5′-AATGGGTTCACAAATCAGCAC-3′
Rat and mouse *VEGFA*	Forward	5′-TGCTGTACCTCCACCATGC-3′
	Reverse	5′-GATGTCCACCAGGGTCTCAA-3′
Rat *GLUT1*	Forward	5′-CCCTGCAGTTCGGCTATAA-3′
	Reverse	5′-AGTGTGGTGAGTGTGGTGGA-3′
Mouse *GLUT1*	Forward	5′-TTATTGCCCAGGTGTTTGG-3′
	Reverse	5′-GTTACGATTGATGAGCAGGAAG-3′
Rat *ACTB*	Forward	5′-TGACGTTGACATCCGTAAAGAC-3′
	Reverse	5′-AGAGCCACCAATCCACACA-3′
Mouse *ACTB*	Forward	5′-TGACAGGATGCAGAAGGAGA-3′
	Reverse	5′-GCTGGAAGGTGGACAGTGAG-3′

### NF-κB inhibition and HIF-1α knockdown

To inhibit NF-κB activity, 10 nM of Bay 11–7082 (Wako Pure Chemical Industries, Osaka, Japan), a selective IκB kinase inhibitor, was administered 1 h before the hypoxic challenge in MLE15 cells. For HIF-1α knockdown, MLE15 cells were transfected with HIF-1α small interfering RNA (siRNA) (siGENOME Mouse Hif1a siRNA SMARTpool, Thermo Fisher Scientific) by using Lipofectamine RNAiMAX (Life Technologies Japan). Control cells were transfected with nontarget siRNA (siGENOME Non-Targeting siRNA Pool #1, Thermo Fisher Scientific). Before the experiments, the cells were incubated in a transfection mixture for 24 h followed by incubation in FBS-free medium for 12 h.

### Statistical analysis

Data are presented as means ± SEM or medians (interquartile ranges). Two-way repeated-measures analysis of variance (ANOVA) followed by Student’s *t* test with Bonferroni correction was performed to compare the BLV and ULV groups. Histological scores were analyzed with Kruskal–Wallis test followed by Dunn’s multiple comparison test. One-way ANOVA followed by Dunnett’s test was performed to compare the ULV, NTV60 %, and NTV0 % groups. Student’s *t* test was performed to compare cell-culture findings. Two-way ANOVA followed by Student’s *t* test with Bonferroni correction was performed for multiple comparisons of the cell-culture experiments. Cytokine concentrations were analyzed after performing log transformations to adjust the standard deviations. GraphPad Prism 6 (GraphPad Software, La Jolla, CA) was used for all statistical analyses. Statistical significance was set at *p* < 0.05.

## Results

### Mean arterial pressure, peak airway pressure, and arterial blood gas analysis

**Fig. 1:**
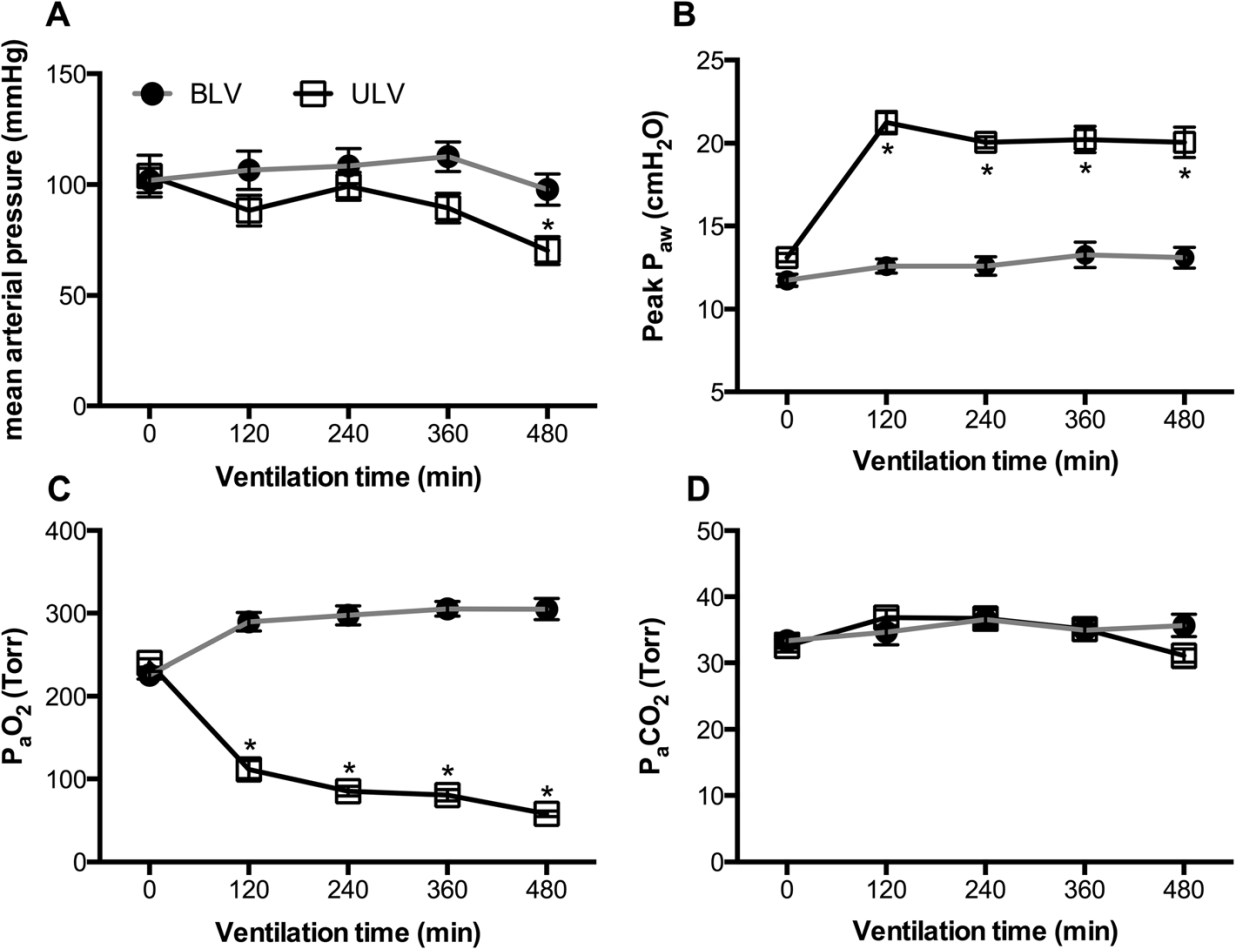
Mean arterial pressure, peak airway pressure, and arterial blood gas analysis. **a** Mean arterial pressure, **b** peak airway pressure, **c** PaO_2_, and **d** PaCO_2_. *p < 0.05 compared with the BLV group (*n* = 8 rats per group). *PaO*_*2*_ arterial partial pressure of oxygen, *PaCO*_*2*_ arterial partial pressure of carbon dioxide, *BLV* bilateral lung ventilation, *ULV* unilateral lung ventilation

MAP was not significantly different between the groups until 360 min; however, it was lower in the ULV group than in the BLV group at 480 min (70 ± 18 vs. 98 ± 20 mmHg, *p* = 0.0444) (Fig. 1a). The peak *P*_aw_ was higher in the ULV group than that in the BLV group (Fig. 1b). Arterial partial pressure of O_2_ (PaO_2_) was significantly higher in the BLV group than that in the ULV group at all the time points after the initiation of ventilation protocols (Fig. 1c). Arterial partial pressure of CO_2_ (PaCO_2_) was not significantly different between these groups (Fig. 1d).

### Inflammatory mediators in lung homogenates

**Fig. 2:**
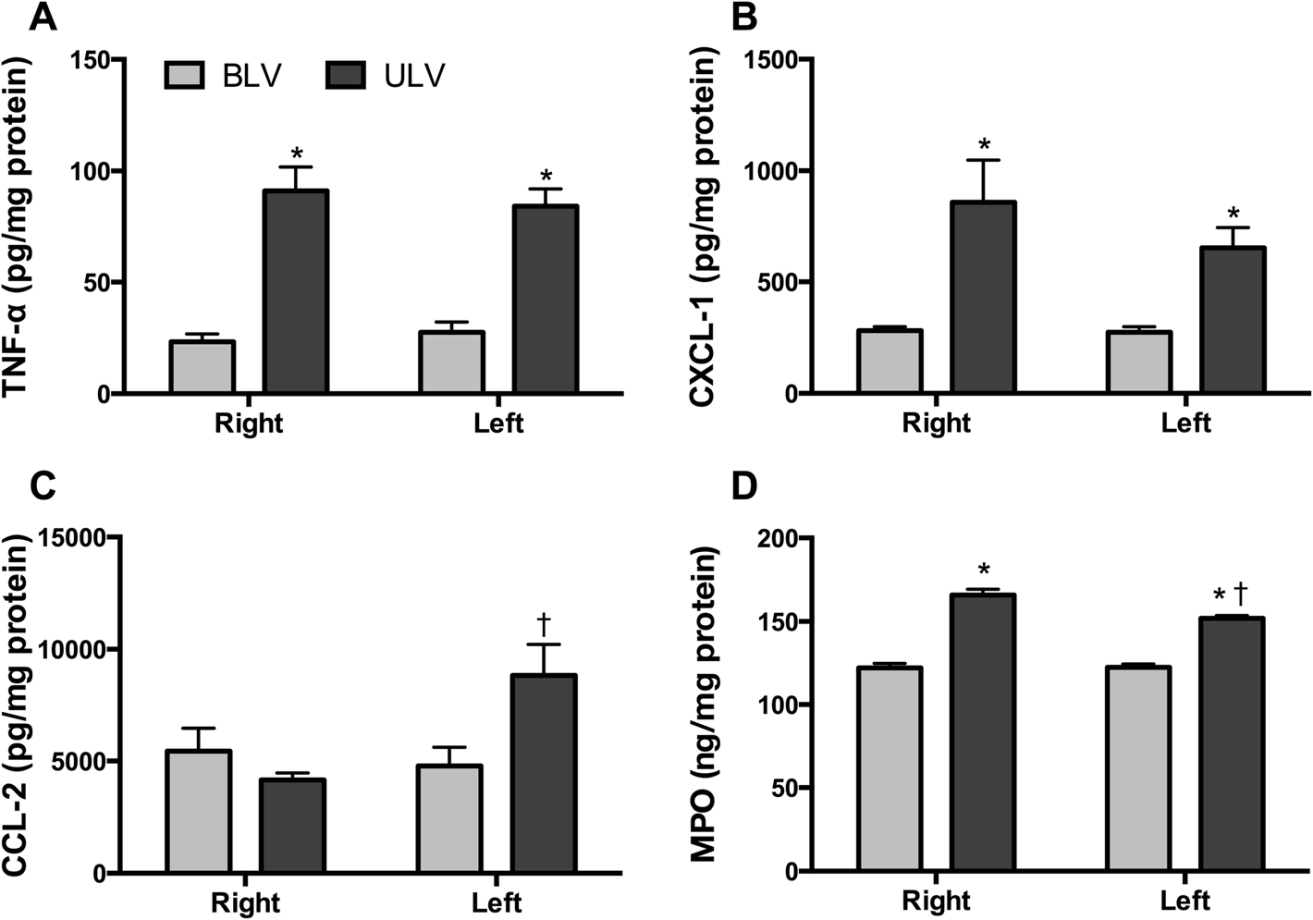
Concentrations of proinflammatory cytokines and MPO in the right (atelectatic) and the left (ventilated with high tidal volume) lung homogenates. **a** TNF-α, **b** CXCL-1, **c** CCL-2, and **d** MPO concentrations were measured by ELISA and normalized to the total protein concentration. *p < 0.05 compared with the BLV group, †p < 0.05 compared with the right lungs (*n* = 8 rats per group). *TNF-α* tumor necrosis factor α, *CXCL-1* chemokine (C-X-C motif) ligand 1, *CCL-2* chemokine (C-C motif) ligand 2, *MPO* myeloperoxidase

TNF-α, CXCL-1, CCL-2, and MPO were detected in lung homogenates of the BLV group (Fig. 2). ULV significantly increased the levels of TNF-α (*F*(1, 14) = 72.87, *p* < 0.0001) (Fig. 2a) and CXCL-1 (*F*(1, 14) = 28.53, *p* < 0.0001) (Fig. 2b) in bilateral lungs. The degrees of increases in TNF-α and CXCL-1 were similar in the bilateral lungs of the ULV group (effect of interaction *F*(1, 14) = 0.9889, *p* = 0.3669, and *F*(1, 14) = 0.8173, *p* = 0.3813, respectively). No significant change in the CCL-2 level was observed between the two groups (Fig. 2c). The CCL-2 level was significantly increased in the left HTV-ventilated lungs compared to the right atelectatic lung in the ULV group (*p* = 0.0365). Consistent with the change in the level of CXCL-1: a potent neutrophil chemoattractant, the concentration of MPO: an indicator of neutrophil accumulation, was significantly higher in the bilateral lungs of the ULV group compared to that in the BLV group (*p* < 0.0001) (Fig. 2d). Moreover, the concentration of MPO was significantly higher in the right atelectatic lung than that in the left HTV-ventilated lung in the ULV group (*p* = 0.0022).

### Histological examination

**Fig. 3:**
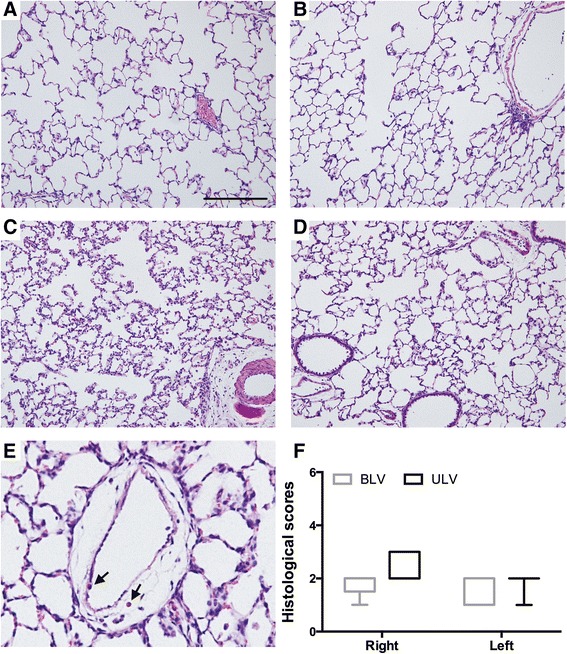
Histological assessment of lung sections after 8 h of ventilation. **a–e** Representative images of lung sections stained with hematoxylin and eosin. **a** Right and **b** left lung sections from the BLV group and **c** right (atelectatic) and **d** left (ventilated with high tidal volume) lung sections from the ULV group. **e** High-magnification images of perivascular edema from right lung sections of the ULV group. *Arrows* indicate neutrophils adhering to tunica intima and infiltrating to the perivascular edema. Scale bar = 200 μm. **f** Histological scores. *BLV* bilateral lung ventilation, *ULV* unilateral lung ventilation

In the BLV group, almost-normal structures were noted in both lungs (Fig. 3a, b). Perivascular and peribronchial edema, leukocytic infiltration of alveolar spaces, and leukocyte stasis and attachment to the tunica intima of the vascular walls were modestly observed in the ULV group, especially in the right atelectatic lung (Fig. 3c, d, e). However, the histological changes were relatively minimal and other indicative features of lung injury, such as formation of hyaline membranes, presence of proteinaceous debris in the alveolar space, or thickening of the alveolar wall [19], were not observed. The histological scores were not significantly different between the BLV and ULV groups (Fig. 3f).

### NF-κB and HIF-1 activities

**Fig. 4:**
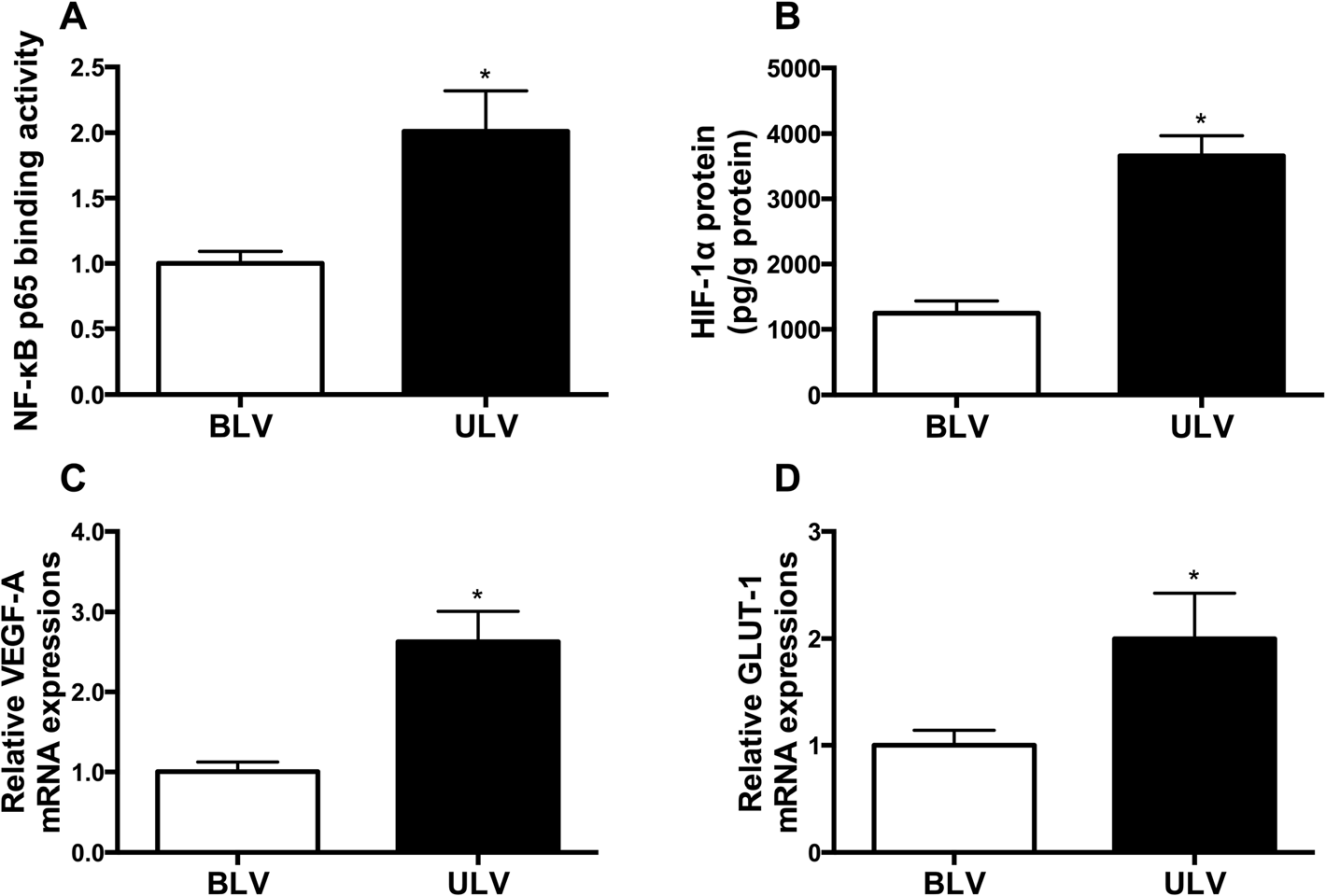
Activities of NF-κB and HIF-1 in right lungs **a** NF-κB p65 binding activities and **b** HIF-1α protein concentrations in right lung homogenates quantified by ELISA. **c** *VEGFA* and **d** *GLUT1* mRNA levels in right lung homogenates measured by qPCR. *p < 0.05 compared with the BLV group (*n* = 8 samples per group). *NF-κB* nuclear factor-κB, *HIF-1* hypoxia-inducible factor 1, *VEGFA* vascular endothelial growth factor A, *GLUT1* glucose transporter-1, *BLV* bilateral lung ventilation, *ULV* unilateral lung ventilation

NF-κB p65 binding activities are increased in the atelectatic lungs of the ULV group compared with the BLV group (2.01 ± 0.30 vs. 1.00 ± 0.09, *p* = 0.0070) (Fig. 4a). HIF-1α concentrations (3658 ± 311.6 vs*.* 1249 ± 186.7 pg/g protein, *p* < 0.0001) (Fig. 4b) and mRNA levels of HIF-1 downstream genes *VEGFA* (2.630 ± 0.3778 vs*.* 1.000 ± 0.1227, *p* = 0.0011) (Fig. 4c) and *GLUT1* (2.000 ± 0.4276 vs*.* 1.000 ± 0.1423, *p* = 0.0436) (Fig. 4d) were also significantly increased in these lungs of the ULV group.

### Effects of NTV ventilation with 60 % O_2_ or 100 % N_2_ on lung inflammation

**Fig. 5:**
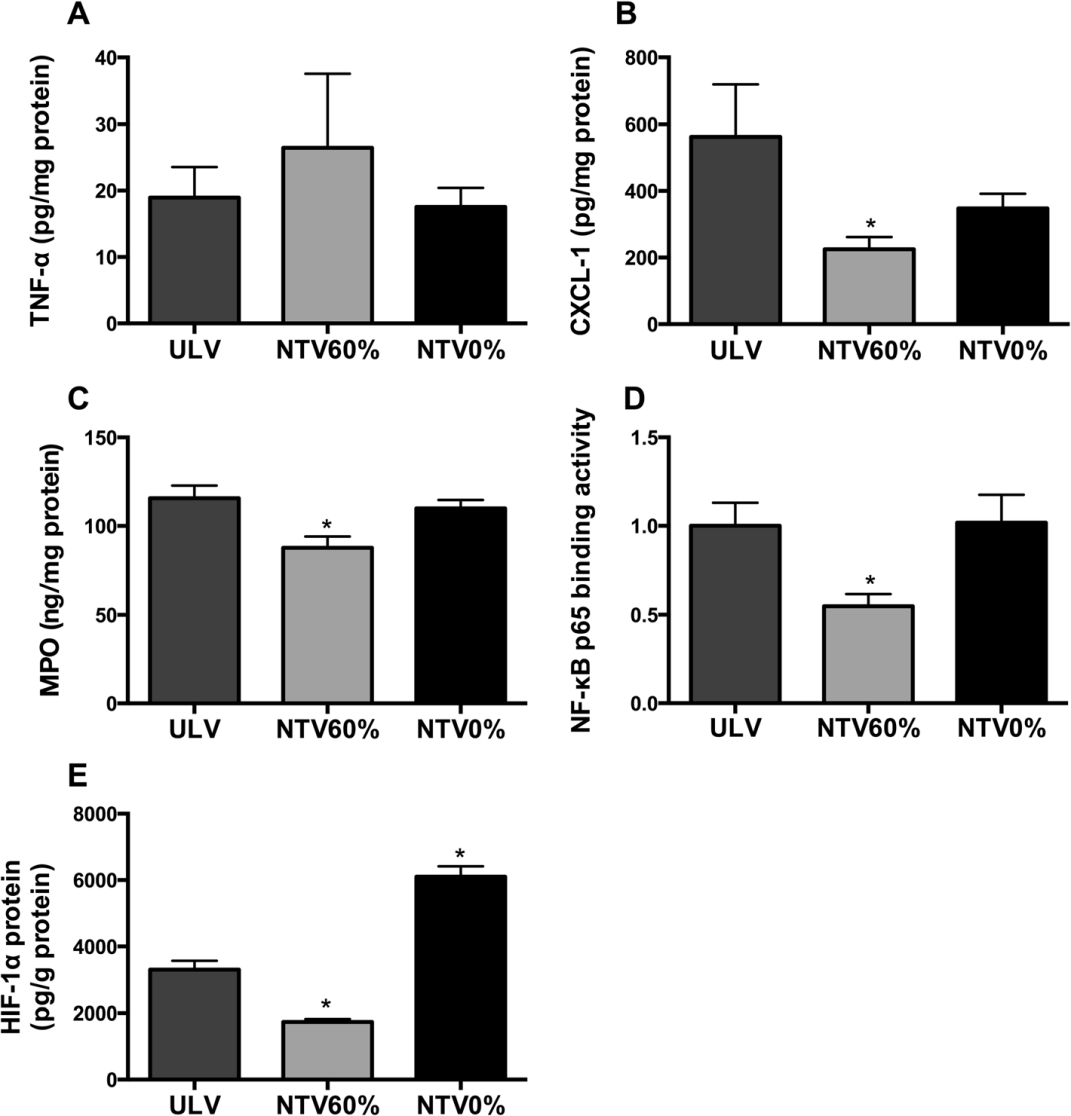
Concentrations of proinflammatory cytokines and MPO and activity of NF-κB and HIF-1α in right lung homogenates. **a** TNF-α, **b** CXCL-1, and **c** MPO concentrations and **d** NF-κB p65 binding activities and **e** HIF-1α concentrations in right lung homogenates were measured by ELISA and normalized to the total protein concentration. *p < 0.05 compared with the ULV group (*n* = 5 rats per group). *TNF-α* tumor necrosis factor α, *CXCL-1* chemokine (C-X-C motif) ligand 1, *MPO* myeloperoxidase, *NF-κB* nuclear factor-κB, *HIF-1* hypoxia-inducible factor 1, *ULV* unilateral lung ventilation, *NTV* normal tidal volume

NTV ventilation of the right lungs with 60 % O_2_ or 100 % N_2_ did not significantly alter the TNF-α level compared to the ULV group (Fig. 5a). NTV ventilation with 60 % O_2_ significantly attenuated the increase in CXCL-1 (562.5 ± 156.7 vs*.* 225.1 ± 36.31 pg/mg protein, *p* = 0.0243) and MPO (115.6 ± 7.212 vs*.* 87.73 ± 6.375 ng/mg protein, *p* = 0.0148) levels observed in the ULV group (Fig. 5b, c). No significant changes in CXCL-1 (562.5 ± 156.7 vs*.* 347.4 ± 44.36 pg/mg protein, *p* = 0.3644) and MPO (115.6 ± 7.212 vs. 109.9 ± 4.834 ng/mg protein, *p* = 0.7445) concentrations were observed between the atelectatic lungs of the ULV group and the lungs ventilated with 100 % N_2_. NF-κB p65 binding activity and the concentration of HIF-α were decreased in the NTV60 % group compared to the ULV group (Fig. 5d, e). The concentration of HIF-1α was significantly increased in the NTV0 % group compared to that in the ULV group.

### Cytokine secretion in vitro

**Fig. 6:**
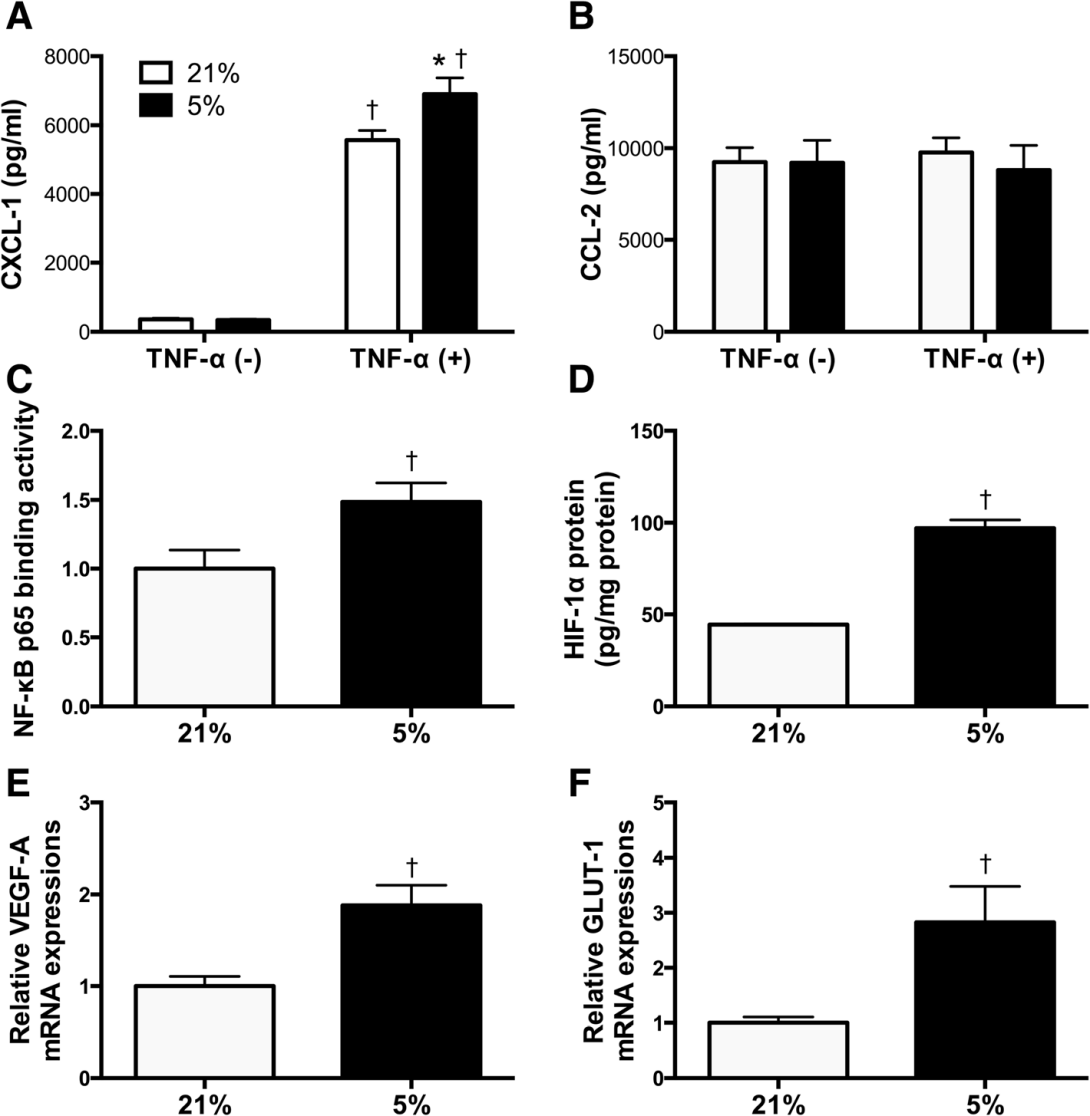
Effect of hypoxia on cytokine secretion from MLE15 and MH-S cells and activities of NF-κB and HIF-1α in MLE15 cells. **a**, **b** Cells were cultured in 21 % O_2_ (*white bar*) or 5 % O_2_ (*black bar*) with or without TNF-α (500 pg/mL). **a** CXCL-1 secreted from MLE15 cells and **b** CCL-2 secreted from MH-S cells. (*n* = 6 samples per group). **c–f** MLE15 cells were cultured in 21 % O_2_ (*white bar*) or 5 % O_2_ (*black bar*) with TNF-α (500 pg/mL). **c** NF-κB p65 binding activities (*n* = 5 samples per group) and **d** HIF-1α concentrations in MLE15 cells (*n* = 4 samples per group). **e** *VEGFA* and **f** *GLUT1* mRNA levels in MLE15 cells (*n* = 5 samples per group). *p < 0.05 compared with the 21 % O_2_ group, †*p* < 0.05 compared with the group without TNF-α. *TNF-α* tumor necrosis factor α, *CXCL-1* chemokine (C-X-C motif) ligand 1, *CCL-2* chemokine (C-C motif) ligand 2, *NF-κB* nuclear factor-κB, *HIF-1* hypoxia-inducible factor 1, *VEGFA* vascular endothelial growth factor A, *GLUT1* glucose transporter-1

Next, we investigated the effects of hypoxia on cytokine secretion from murine lung epithelial (MLE15) and alveolar macrophage (MH-S) cells. We treated these cells with TNF-α because TNF-α is known as an essential mediator for the pathogenesis of VALI [20, 21] and the concentration of TNF-α was not affected by NTV ventilation with 60 % O_2_ in our in vivo experiment. MLE15 cells cultured in 5 % O_2_ secreted a significantly higher concentration of CXCL-1 than those cultured in 21 % O_2_ after treatment with TNF-α (5568 ± 279.7 vs*.*6897 ± 475.7 pg/mL, *p* = 0.012) (Fig. 6a); no significant difference was noted in MLE15 cells not treated with TNF-α. Further, the concentration of CCL-2 secreted from MH-S cells was not significantly different in any culture condition (Fig. 6b).

TNF-α was not detected in the supernatants of MLE15 or MH-S cells, and CXCL-1 was not detected in the supernatants of MH-S cells. CCL-2 was detected in MLE15 cell supernatants only after TNF-α treatment and at a much lower level than that observed in the MH-S cell supernatants (data not shown).

Exposure to 5 % O_2_ significantly increased the binding activity of NF-κB p65 in MLE15 cells cultured with TNF-α (1.49 ± 0.14 in 5 % O_2_ vs. 1.00 ± 0.14 in 21 % O_2_, *p* = 0.0367) (Fig. 6c). HIF-1α concentrations (97.1 ± 4.5 pg/mg protein in 5 % O_2_ vs. 44.5 ± 0.31 pg/mg protein in 21 % O_2_, *p* < 0.0001) (Fig. 6d) and *VEGFA* (1.88 ± 0.22 in 5 % O_2_ vs. 1.00 ± 0.11 in 21 % O_2_, *p* = 0.0071) (Fig. 6e) and *GLUT1* (2.83 ± 0.66 in 5 % O_2_ vs. 1.00 ± 0.11 in 21 % O_2_, *p* = 0.0252) (Fig. 6f) mRNA expressions also increased in MLE15 cells cultured with TNF-α under the hypoxic condition.

### Roles of NF-κB and HIF-1 in hypoxia-induced inflammatory responses

**Fig. 7:**
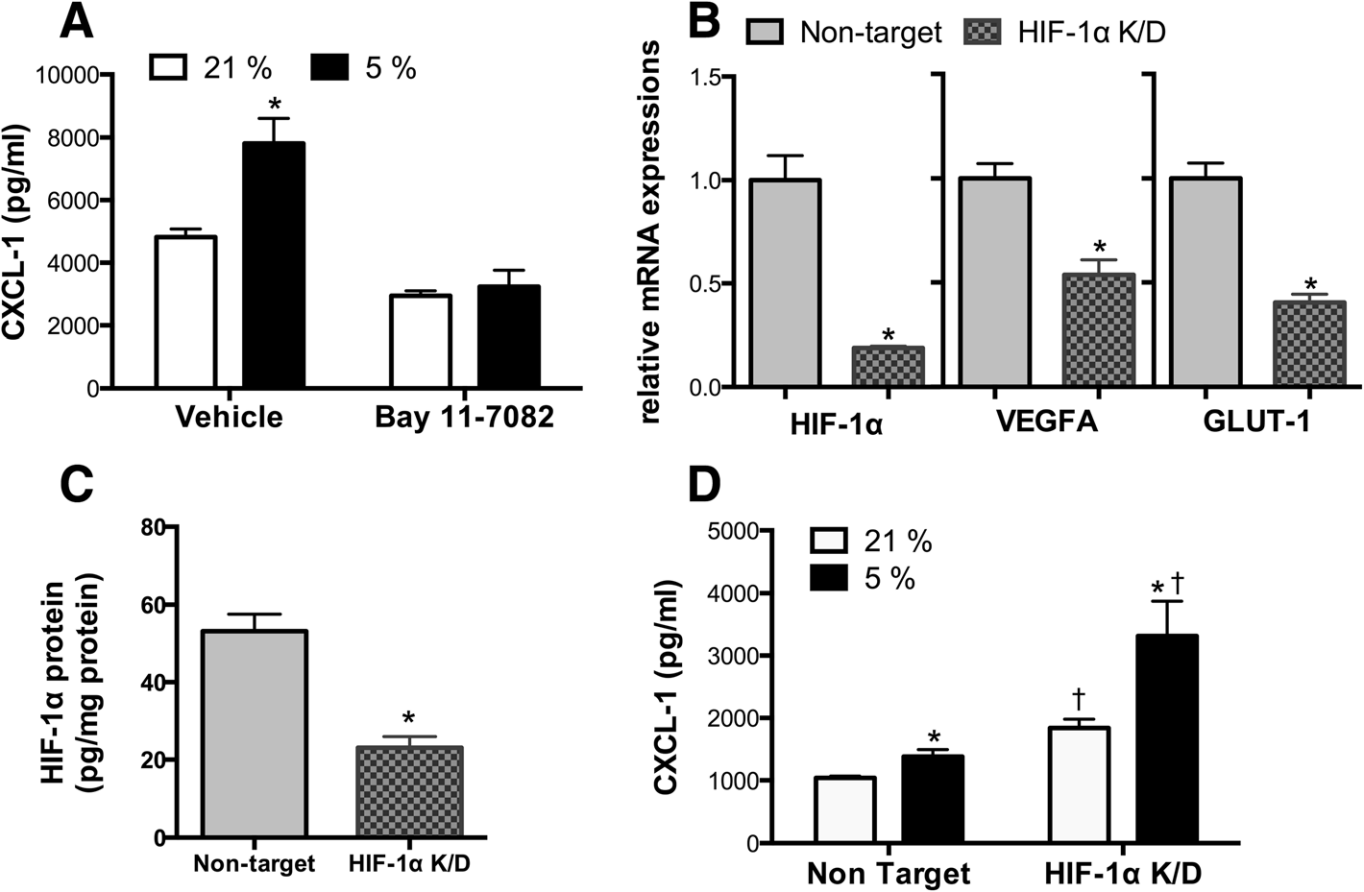
Effects of NF-κB inhibition and HIF-1α knockdown on cytokine secretion from MLE15 cells. Cells were cultured in 21 % O_2_ (*white bar*) or 5 % O_2_ (*black bar*) with TNF-α (500 pg/mL). **a** CXCL-1 secretion from MLE15 cells cultured with 500 pg/mL TNF-α after treatment with Bay 11–7082 or vehicle. *p < 0.05 compared with the 21 % O_2_ group (*n* = 6 samples per group). **b** *HIF1A*, *VEGFA*, and *GLUT1* mRNA levels and **c** HIF-1α protein concentrations in MLE15 cells transfected with HIF-1α or nontarget siRNA. *p < 0.05 compared with the nontarget groups (*n* = 5 samples per group). **d** CXCL-1 secretion from MLE15 cells cultured with 500 pg/mL TNF-α after transfection with HIF-1α or nontarget siRNA. *p < 0.05 compared with the 21 % O_2_ group, †*p* < 0.05 compared with the nontarget group (*n* = 6 samples per group). *NF-κB* nuclear factor-κB, *HIF-1* hypoxia-inducible factor 1, *VEGFA* vascular endothelial growth factor A, *GLUT1* glucose transporter-1, *K/D* knockdown, *CXCL-1* chemokine (C-X-C motif) ligand 1

NF-κB inhibition by Bay 11–7082 abolished the hypoxia-induced upregulation of CXCL-1 in MLE15 cells cultured with TNF-α (two-way ANOVA: effect of oxygen concentration *F*(1, 20) = 10.53, *p* = 0.0041; effect of NF-κB inhibition *F*(1, 20) = 40.73, *p* < 0.0001; interaction *F*(1, 20) = 7.122, *p* = 0.0148; post hoc analysis: 21 vs*.* 5 % O_2_ in vehicle group, *p* = 0.0018; 21 vs.5 % O_2_ in Bay 11–7082 group, *p* > 0.9999) (Fig. 7a). HIF-1α knockdown effectively decreased *HIF1A*, *VEGFA*, and *GLUT1* mRNA levels (Fig. 7b) and the HIF-1α protein concentration (Fig. 7c). However, it increased CXCL-1 secretion from MLE15 cells cultured with TNF-α (two-way ANOVA: effect of oxygen concentration *F*(1, 20) = 9.401, *p* = 0.0061; effect of HIF-1α knockdown *F*(1, 20) = 21.38, *p* = 0.0002; interaction *F*(1, 20) = 3.665, *p* = 0.0700) (Fig. 7d).

## Discussion

In this study, we demonstrated increases of proinflammatory cytokines and neutrophil accumulation in nonventilated atelectatic lungs during ULV. Improvement in alveolar hypoxia by NTV ventilation with 60 % O_2_ significantly attenuated these inflammatory responses; however, NTV ventilation with 0 % O_2_ did not. These results suggest that alveolar hypoxia in atelectasis causes inflammation and may contribute to VALI. Moreover, our in vitro results suggest that hypoxia-induced NF-κB activation is responsible for increased CXCL-1 secretion from lung epithelial cells in atelectatic lungs, while HIF-1 negatively regulates CXCL-1 secretion under hypoxic conditions.

The concentrations of inflammatory cytokines in the atelectatic lungs were comparable to those in the HTV-ventilated lungs. Moreover, MPO levels were significantly higher in the atelectatic lungs than in the HTV-ventilated lungs. These data suggest that the inflammatory responses in the atelectatic lungs were caused by atelectasis rather than indirectly by contralateral HTV ventilation. There are conflicting reports regarding the topography of lung inflammation during mechanical ventilation. A previous report has demonstrated predominant inflammation in the aerated overinflated regions in rats with surfactant depletion [1]. We used a relatively lower tidal volume and peak *P*_aw_ compared with that study [1], which used 24 mL/kg tidal ventilation without limitation of peak *P*_aw_. Therefore, the injurious effects of HTV and overinflation may have been minimal in the present study. Recently, it has been reported that injurious ventilation with 0 cmH_2_O PEEP and limitation of peak *P*_aw_ induces predominant inflammation in the dependent lungs of sheep with LPS-induced lung injury [22]. It is possible that uneven mechanical ventilation with moderate HTV ventilation of the nondependent lung, which is often encountered in clinical settings, may cause predominant inflammation in the dependent atelectatic lung.

Our ULV protocol did not cause severe lung histological damage, and the alveolar integrity was maintained. These findings are consistent with our previous report [18] which demonstrated that HTV ventilation (24 mL/kg) does not cause severe lung injury without concomitant systemic inflammation induced by hepatic ischemia–reperfusion in rats. The inflammatory responses that we observed are possible early events of VALI. These inflammatory responses may not only be reversible [23] but may also lead to severe injury when other multiple injurious events have occurred.

We used 60 % O_2_ for nonhypoxic condition because 60 % O_2_ does not show evident toxicity in healthy animals and is considered to be clinically safe in humans [24]. Meanwhile, it has been reported that exposure of mice with LPS-induced lung injury to 60 % O_2_ exaggerates the lung injury [25]. In the present study, bilateral lung ventilation in healthy rats with 60 % O_2_ for 8 h did not cause evident inflammation and injury. Moreover, we demonstrated that NTV ventilation with 60 % O_2_ significantly attenuated the inflammation in the atelectatic lung; however, anoxic ventilation did not. Although hyperoxia is harmful to lungs, alveolar hypoxia in the nonaerated, atelectatic lungs also seems to be a cause of pulmonary inflammation and may contribute to VALI.

The CXCL-1 concentration was not statistically different between the atelectatic lung and the NTV-ventilated lung with 0 % O_2_. However, the CXCL-1 concentration tended to decrease in the NTV0 % group. There are some possible explanations for this apparent decrease. First, the HIF-1 concentration was elevated in the right lungs of the NTV0 % group compared with those of the ULV group, although there was no difference in NF-kB DNA-binding activity. The anti-inflammatory effects of HIF-1 may have contributed to the decreased level of CXCL-1 in the NTV0 % group. Second, the mechanical inflation effect of NTV ventilation may have attenuated the CXCL-1 elevation. Although, the precise mechanisms are unclear, mechanical inflation can attenuate lung injury induced by ischemia–reperfusion [26, 27]. However, alveolar hypoxia appears to be the main cause of inflammation in atelectatic lungs because NTV ventilation with 60 % O_2_ attenuated the inflammation more prominently than those with 0 % O_2_ did_,_

The open-chest differential-ventilation rat model offers some advantages. First, we could investigate the effects of atelectasis in nonventilated lungs without repetitive shear stress caused by alveolar collapse and reopening [3–5]. Therefore, we could demonstrate that alveolar hypoxia is one of the main causes of lung inflammation in atelectatic lungs. Second, in a previous study of the effects of alveolar hypoxia on lung inflammation [28], mice exposed to a hypoxic condition developed systemic hypoxemia. Systemic hypoxemia may have different effects to those of alveolar hypoxia. We could induce alveolar hypoxia, which is clinically more common in lung injury, and evaluate its effects without severe systemic hypoxemia.

Our in vitro data suggest that alveolar epithelial cells contribute to hypoxia-induced inflammation in lungs. In contrast, we did not detect hypoxia-induced inflammatory responses in alveolar macrophages in vitro. The absence of changes of CCL-2 concentration in the atelectatic lung is consistent with the in vitro data. Previous studies have demonstrated that hypoxia-induced CCL-2 release from alveolar macrophages leads to systemic inflammation [29, 30]. According to these reports, CCL-2 release from alveolar macrophages occurs early (15 min to 1 h) after exposure to hypoxia. Therefore, it is possible that the evaluation time points we used in this study did not allow us to detect increased CCL-2 release from alveolar macrophages.

The NF-κB pathway is required for hypoxia-induced inflammatory gene expression [31], and inhibition of NF-κB activity attenuates hypobaric hypoxia-induced lung edema [32]. Our finding that NF-κB activation is responsible for hypoxia-induced increase in CXCL-1 secretion from lung epithelial cells is consistent with these previous studies [31, 32]. Regarding HIF-1, HIF-1α is essential for myeloid cell-mediated inflammation [15], and HIF-1α deletion from the myeloid cell lineage attenuates lipopolysaccharide-induced sepsis in mice and raises survival rates [17]. *HIF1A* mRNA expression is increased in shock patients [33], and decreased *HIF1A* mRNA expression in patients with sepsis may be associated with depressed immune function [34]. Moreover, drugs inhibiting HIF-1 activities attenuate lipopolysaccharide-induced lung injury [35, 36]. However, some reports suggest that HIF-1 has anti-inflammatory effects. Targeted deletion of HIF-1α in T lymphocytes in mice with bacterial sepsis leads to higher levels of proinflammatory cytokines and stronger antibacterial capacities [37]. HIF-1 activation in lung epithelial cells protects against VALI through optimization of carbohydrate metabolism [38]. Our finding that HIF-1 knockdown augments hypoxia-induced increase in CXCL-1 secretion from lung epithelial cells suggests that HIF-1 activation in atelectatic lungs may be a protective response to hypoxia and prevents lung inflammation and injury. Further investigations on the roles of HIF-1 in lung injury are warranted.

Alveolar hypoxia in the atelectatic lung may be a novel therapeutic target for VALI. Our results support the rationale for open lung approaches, including application of higher PEEP and lung recruitment maneuvers. The clinical benefits of the open lung approach [39] may contribute to the improvement of alveolar hypoxia. On the other hand, the increase of the inspired O_2_ fraction may also increase alveolar oxygen tension even when atelectasis exists [40]. Although it is possible that higher oxygen concentration shows toxic effects, increasing inspired O_2_ fraction may be able to attenuate hypoxia-induced inflammatory responses in the atelectatic lung region. Clinical trials to investigate optimal strategies to adjust the inspired O_2_ fraction are thus warranted.

This study has several limitations. First, the differential-ventilation model may not accurately reflect uneven mechanical ventilation in clinical settings, although it has some advantages as described previously. Second, although the systemic circulation was not severely impaired by ULV, we could not control pulmonary circulation during ULV. Pulmonary hypertension in response to alveolar hypoxia in atelectatic lungs may have contributed to lung injury [40, 41]. However, our in vitro observation that hypoxia significantly increases CXCL-1 secretion from lung epithelial cells strongly suggests that alveolar hypoxia is one of the main causes of inflammatory responses in atelectatic lungs. Third, the follow-up period was up to 8 h, and we did not evaluate any functional outcomes, such as mortality. Further investigations are needed to evaluate long-term effects of alveolar hypoxia in the atelectatic lungs on VALI. Fourth, we evaluated the contributions of NF-κB and HIF-1 to hypoxia-induced lung inflammation only in a lung epithelial cell line and by considering a single inflammatory mediator in vitro. Although, the critical role of CXCL-1 for the pathogenesis of VALI has been reported [42], further studies using animals and another cell types are needed.

## Conclusions

Atelectasis causes alveolar hypoxia-induced inflammatory responses including NF-κB-dependent CXCL-1 secretion from lung epithelial cells. HIF-1 activation in lung epithelial cells is an anti-inflammatory response to alveolar hypoxia in atelectatic lungs. Modulation of HIF-1α and NF-κB in lung epithelial cells may present therapeutic approaches for VALI.

### Key messages

- Atelectasis causes alveolar hypoxia-induced inflammatory responses including NF-κB-dependent CXCL-1 secretion from lung epithelial cells.
- HIF-1 activation in lung epithelial cells is an anti-inflammatory response to alveolar hypoxia in atelectatic lungs.

## Abbreviations

*ACTB*: β-actin
ANOVA: analysis of variance
BLV: bilateral lung ventilation
CCL-2: chemokine (C-C motif) ligand 2
CXCL-1: chemokine (C-X-C motif) ligand 1
ELISA: enzyme-linked immunosorbent assay
FBS: fetal bovine serum
*GLUT1*: glucose transporter-1
HIF-1: hypoxia-inducible factor 1
HTV: high tidal volume
IκB: inhibitors of κB
IL: interleukin
MAP: mean arterial pressure
MPO: myeloperoxidase
NF-κB: nuclear factor-κB
NTV: normal tidal volume
PaCO_2_: arterial partial pressure of carbon dioxide
PaO_2_: arterial partial pressure of oxygen
*P*_aw_: airway pressure
PEEP: positive end-expiratory pressure
PHD: prolyl hydroxylase
qPCR: quantitative polymerase chain reaction
siRNA: small interfering RNA
TNF-α: tumor necrosis factor α
ULV: unilateral lung ventilation
VALI: ventilator-associated lung injury
*VEGFA*: vascular endothelial growth factor A
